# Cooperating elephants mitigate competition until the stakes get too high

**DOI:** 10.1371/journal.pbio.3001391

**Published:** 2021-09-28

**Authors:** Li-Li Li, Joshua M. Plotnik, Shang-Wen Xia, Estelle Meaux, Rui-Chang Quan

**Affiliations:** 1 Center for Integrative Conservation, Xishuangbanna Tropical Botanical Garden, Chinese Academy of Sciences, Mengla, Yunnan, China and Southeast Asia Biodiversity Research Institute, Chinese Academy of Sciences, Nay Pyi Taw, Myanmar; 2 University of Chinese Academy of Sciences, Beijing, China; 3 Department of Psychology, Hunter College, City University of New York, New York, New York, United States of America; 4 Department of Psychology, The Graduate Center, City University of New York, New York, New York, United States of America; 5 CAS Key Laboratory of Tropical Forest Ecology, Xishuangbanna Tropical Botanical Garden, Chinese Academy of Sciences, Mengla, Yunnan, China; 6 Guangxi Key Laboratory of Forest Ecology and Conservation, College of Forestry, Guangxi University, Nanning, Guangxi, China; 7 Center of Conservation Biology, Core Botanical Gardens, Chinese Academy of Sciences, Mengla, Yunnan, China; University of St Andrews, UNITED KINGDOM

## Abstract

Cooperation is ubiquitous in the animal kingdom as it aims to maximize benefits through joint action. Selection, however, may also favor competitive behaviors that could violate cooperation. How animals mitigate competition is hotly debated, with particular interest in primates and little attention paid thus far to nonprimates. Using a loose-string pulling apparatus, we explored cooperative and competitive behavior, as well as mitigation of the latter, in semi-wild Asian elephants (*Elephas maximus*). Our results showed that elephants first maintained a very high cooperation rate (average = 80.8% across 45 sessions). Elephants applied “block,” “fight back,” “leave,” “move side,” and “submission” as mitigation strategies and adjusted these strategies according to their affiliation and rank difference with competition initiators. They usually applied a “fight back” mitigation strategy as a sanction when competition initiators were low ranking or when they had a close affiliation, but were submissive if the initiators were high ranking or when they were not closely affiliated. However, when the food reward was limited, the costly competitive behaviors (“monopoly” and “fight”) increased significantly, leading to a rapid breakdown in cooperation. The instability of elephant cooperation as a result of benefit reduction mirrors that of human society, suggesting that similar fundamental principles may underlie the evolution of cooperation across species.

## Abstract 2

This study shows that in a task requiring coordinated pulling, elephants compete for access to food but work to mitigate competition in order to maintain cooperation. If the cost of competition becomes too high, however, cooperation breaks down entirely. This behavior mirrors that seen in humans and other great apes, suggesting that certain cooperative mechanisms are not unique to primates.

## Introduction

Cooperation—joint action for common benefits—is not unusual among social animals [1] and can usually increase the fitness of cooperators [2]. In harsh and unpredictable environments, organisms can increase their survival rate through cooperation, such as when some herbivores group together to defend against predators or wolves cooperate to hunt prey [3–6]. There are 4 evolutionary mechanisms proposed for the emergence of cooperation: kin selection (i.e., cooperation evolved to increase inclusive fitness) [7,8], reciprocity (individuals help each other in a tit-for-tat fashion to provide measured benefits) [9–11], by-product mutualism (working together to obtain mutual benefits, usually at the same time) [6,12], and group selection (where a group of cooperators outcompete a group of defectors) [1,13]. Although cooperation may evolve within groups to promote individual-level fitness, it is not always stable. Often, competitive behaviors such as freeloading (or cheating) emerge when individuals increase their own fitness by accepting benefits from others without providing them in return (i.e., obtaining all the benefits without incurring any of the costs of cooperation [14]). These competitions could undermine long-term cooperative relationships [15], even leading to their breakdown and thus decreasing overall payoffs between group members [16]. We know that cooperation is a widespread social construct in the animal kingdom, and thus it has been hypothesized that mitigation mechanisms have co-emerged to manage competition [17]. However, research in this area has thus far been surprisingly limited.

According to the “emotional reactivity hypothesis,” those who are tolerant of conspecifics during social problem-solving perform better in cooperation [18,19]. For instance, Hare and colleagues [20] argued that because bonobos were more tolerant than chimpanzees (as measured during co-feeding), bonobos (*Pan paniscus*) succeeded at higher rates than chimpanzees (*Pan troglodytes*) in cooperation tasks in which the food could be monopolized, and thus competition was more likely [21]. In a social group, if subordinate individuals are not sufficiently tolerant of conspecifics, they may avoid dominants or potential within-group competition and thus lose opportunities for cooperation. Therefore, the presence of tolerant behavior may help predict whether cooperation will be maintained within a social group [20,22,23].

In cooperative situations where competition emerges, however, continuous tolerance may reduce cooperative benefits by preventing “cheated” individuals from responding to competition. Thus, other mitigation strategies may be needed to maintain stable levels of cooperation within groups. Humans seem to have developed extraordinarily flexible strategies for maintaining cooperation in highly competitive environments [24]. These strategies include using sanctions such as punishment for cheating or defecting [25], selecting partners based on previous interactions [26], building reputations or making decisions based on the reputations of others [27], and policing [28]. Using these mitigation strategies allows humans to maintain cooperation, specifically between nonkin. Humans seem to be unique in that cooperation can occur and be maintained both within and across groups (even globally), which may distinguish us from other animals [24,29]. Although human cooperation may be more complex across groups, some scientists hypothesize that animals may also be efficient at controlling competition. For instance, chimpanzees use direct protests (i.e., fighting against cheaters), third-party punishments (e.g., dominant chimpanzees punish cheaters even when they are not a party to the immediate conflict), and selective partner choice (i.e., choosing loyal partners over disloyal ones) to control competition and to maximize cooperation within groups [30–32]. This suggests that nonhuman primates may share some similarities with humans in how they maintain cooperative relationships.

The difference between how humans and nonhuman animals control competition likely has to do with how active individuals are in the decision-making process. Humans apply more active mechanisms, such as punishment [33], rewarding [34], or public reputation building [35] to regulate competition and cooperation, while animals may often use passive mechanisms to reduce competition (e.g., they may indirectly benefit from cooperation and thus reduce competition by working with closely related individuals or by receiving immediate rewards from cooperative behavior) [24]. For instance, when presented with a cooperative task with a clumped food reward that can be monopolized, humans tend to share food to maintain cooperation [34], while bonobos tend to tolerate competition to achieve a high cooperation rate [20]. Remarkably, little research has been done on how nonhuman primates mitigate competition and whether they behave flexibly in competitive situations (but see, e.g., [30]). Such work could improve our understanding of the similarities in the evolutionary processes that shape cooperative behavior in humans and nonhuman animals. In addition, expanding attention beyond the primate taxa through studies of evolutionarily distant, highly cooperative species could support the idea that complex cooperation and the regulation of competition within social groups evolved across species due to similar environmental pressures [36,37]. Indeed, some experimental research has explored cooperative behavior in nonprimate taxa, including parrots [38], hyenas [39], otters [40], and ravens [22]; however, how flexible animals are in maintaining cooperative relationships when faced with competition has not been sufficiently explored.

The Asian elephant (*Elephas maximus*) is an interesting and unique subject for the study of cooperation. They are evolutionarily distinct from primates, highly cooperative, and can be tested in controlled environments in captivity in Southeast Asia due to a long history of taming for work and tourism [41]. Female elephants within a family or bond group will often take care of each other’s calves [42], and bull elephants, after reaching sexual maturity, often form bachelor herds to manage harsh environments [43]. Elephants have shown empathetic tendencies [44,45], and Asian elephants in particular have performed well on a number of cognitive tasks, including relative quantity judgment [46–48], means-end understanding [49], self-recognition [50], olfactory cue following [51], insight [52], and tool use [53]. This flexibility in cognition has also been demonstrated in an experimental, cooperative task with elephants. The cooperative paradigm, the loose-string pulling task originally designed by Hirata and Fuwa [54], and since used in a number of studies with a variety of species (including chimpanzees [21], bonobos [20], otters [40], ravens [22,55], wolves and dogs [56], macaques [32], rooks [57], keas [58,59], and humans [34]), is an elegant yet simple task that requires that 2 animals pull 2 ends of the same rope in order to gain access to an out-of-reach food reward. This design can easily facilitate comparisons across species and is especially useful with elephants because it requires coordination between 2 individuals rather than bruit force or strength. Using this rope-pulling task, Plotnik and colleagues [60] showed that paired elephants could learn to wait for partners in order to get food and to refuse pulling if the partner lacked access to the rope. Some elephants also adopted other strategies to solve the task, like standing on one rope end so that it could not be pulled away while the partner pulled (perhaps a form of freeloading requiring the partner to do all the work for equal reward).

To date, further controlled, experimental studies on elephant cooperation have not been done. Thus, in order to understand how elephants maintain their cooperative relationships when competition can occur, we studied a group of semi-wild Asian elephants in Myanmar by exposing them to an open-access loose-string pulling apparatus. We offered 2 food trays on the apparatus first to observe whether competitive behavior appeared and how the elephants mitigated this behavior across pairings to maintain cooperation. We then reduced 2 food trays to 1 to see if cooperation continued when food could be monopolized [34,61]. In the 1-food tray setting, food was put together on a single tray but divisible. We predicted that, because elephants had the opportunity to choose their own partners, various competitive behaviors, such as competing for either standing place (i.e., where to stand in front of the apparatus), the rope ends, and/or food rewards, would appear under the 2-food tray condition. However, we expected that the elephants would develop strategies to mitigate competition and maintain cooperation relative to their affiliation with and rank difference between competitors. Unlike nonhuman primates, Asian elephants are generalized herbivores that do not hunt in groups or share prey [61,62]. Therefore, we also hypothesized that when only 1 tray of food was available, competition mitigation strategies would fail to overcome the unequal rewarding caused by monopolization of the food, and cooperation would break down.

## Results

### Overall cooperation

**Fig 1:**
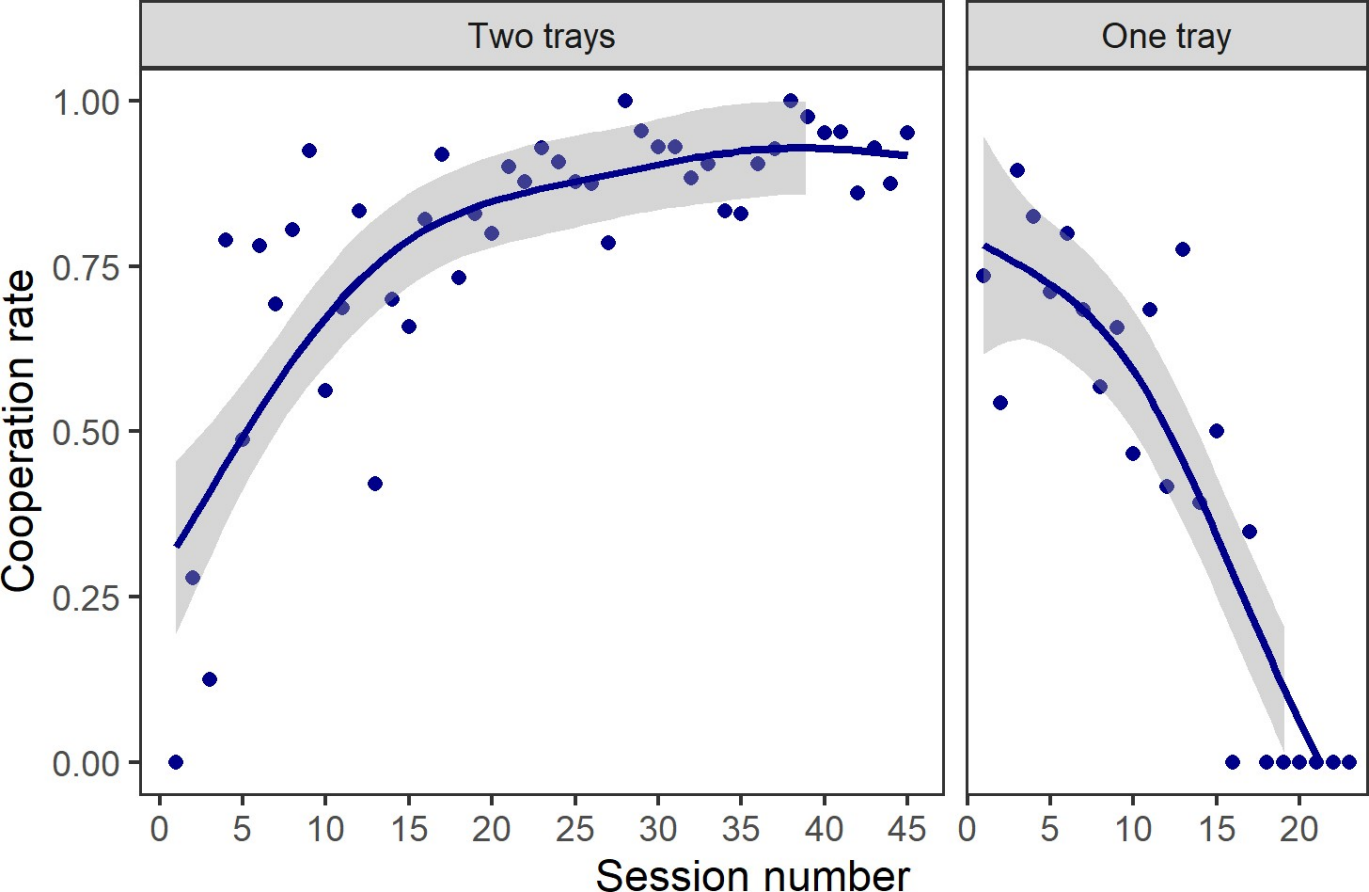
Cooperation rate of Asian elephants in the test under the 2-tray and the 1-tray phase. Cooperation rate was calculated as frequency of successful cooperation divided by frequency of all attempts (i.e., all trials in a session). Each point represents the cooperation rate in each session. Gray areas represent 95% confidence intervals. In the 2-tray Phase I, cooperation was maintained at a high level throughout testing, while cooperation broke down entirely after the 17th session in the 1-tray Phase II. The data used to generate this figure can be found in S1 Data.

Our study involved 9 semi-wild Asian elephants from the Myaing Hay Wun (MHW) Elephant Camp in Taikkyi, Yangon, Myanmar, all owned by the Myanma Timber Enterprise (MTE). Their ages ranged from 6 to 55, with 4 males and 5 females (S1 Table). These 9 elephants all passed initial training (aimed at habituating them to the apparatus and ensuring they could use it to cooperate—see S2 Table for results of training and the Methods section for details on procedure). In testing, a single rope was threaded through and around an out-of-reach table so that each end of the rope needed to be pulled by an elephant in order to get food. All 9 elephants were given access to the table simultaneously in testing to investigate which elephants would cooperate to gain access to the food, whether competition for access to the table would occur, and if and how such competition would be mitigated. In Phase I of testing (i.e., the 2-tray phase, in which 1 tray of food was placed on either end of the table), the elephants successfully cooperated 1,451 times in 1,795 trials across 45 sessions (average cooperation rate = 80.8%; S1 Movie), meaning the cooperation frequency per hour was as high as 32.2. We set 1-minute intervals between trials, and thus it is possible the cooperation tendency could have been even higher. The cooperation rate increased quickly (Fig 1) and remained stable from the 16th session onward (Spearman ρ = 0.75, *P* < 0.001). However, after we changed from the cooperative 2-tray Phase I to the more competitive 1-tray condition in Phase II (in which the food remained divisible but was clumped in 1 tray), cooperation broke down quickly, with the cooperation rate falling to zero and remaining there in 6 consecutive sessions from the 18th session onward (Fig 1; Spearman ρ = −0.86, *P* < 0.001). The frequencies at which each elephant pulled a rope end (i.e., their “contribution”) and consumed food from a tray (i.e., ate their “reward”) in the 2-tray Phase I and 1-tray Phase II are listed in S3 Table. The table shows that dominant elephants participated in the cooperative task most often and also obtained the highest amount of reward across the phases.

### Phase I (2-tray condition): Competition

**Fig 2:**
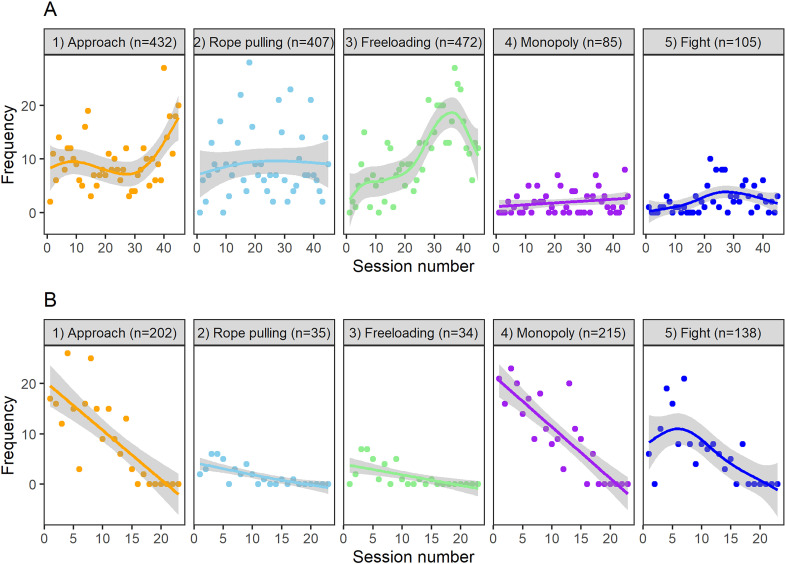
**Change in competition behaviors over time in the 2-tray Phase I (A) and 1-tray Phase II (B).** Each point represents the frequency of a competition behavior in each session. Different colors represent different types of competition behaviors, while gray shading represents 95% confidence intervals. The perceived cost of each type of competition behavior increases from left to right, with approach being least costly and fight being most costly. While the cooperation rate remained stable across the 2-tray Phase I, it decreased rapidly in 1-tray Phase II, which contributed to the decreasing frequency of each competition behavior across Phase II (i.e., when cooperation happened less frequently or not at all, there was less or no food, respectively, for which to compete). The data used to generate this figure can be found in S1 Data.

We recorded 5 types of competition behavior during our experiment. In order of perceived cost to the target elephant (a subjective measure of least to most costly), the behaviors were approach, rope pulling, freeloading, monopoly, and fight (see Table 1 for definitions). Using a generalized additive model, we explored how each type of competition behavior changed over time. During the 2-tray Phase I, the least costly competitive behavior, approach, increased in frequency throughout the phase, but not significantly (R^2^ = 0.059, t = 1.934, *P* = 0.06). The frequency of rope pulling did not change significantly throughout the experiment (R^2^ = −0.018, t = 0.495, *P* = 0.623). The frequency of freeloading changed significantly (increased at the beginning and later decreased; R^2^ = 0.424, t = 5.775, *P* < 0.001). The costliest competition behaviors (monopoly and fight) were rarely observed in Phase I (Fig 2A). The frequency of monopoly did not change significantly across the 2-tray phase (R^2^ = 0.02, t = 1.376, *P* = 0.176), while the frequency of fight changed significantly over time (R^2^ = 0.07, t = 2.072, *P* = 0.044).

**Table 1:** Competitive behaviors of elephants and behavior definitions.

Competitive behaviors	Definition	Potential cost to the target elephant
Approach	When one elephant stands in front of the rope ends, another elephant walks toward them, in order to gain access to the rope or food.	Position (cost is lowest)
Rope pulling	Two elephants pull one end of the rope together.	Rope (cost is low)
Freeloading	Two elephants pull the 2 rope ends, and a third elephant takes the food reward without pulling the rope.	Part of food (cost is low)
Monopoly	Elephant takes all of the food reward on the table.	All food (cost is high)
Fight	Elephant uses the tusk or head to push or puts their trunk over another elephant near the apparatus, a behavior that indicates dominance/higher social ranking.	Physical injury (cost is highest)

### Phase I (2-tray condition): Mitigation strategies

In the 2-tray condition in which food rewards were dispersed, we recorded tolerance percentages (i.e., the frequency of “no response” by an elephant after a conspecific displayed a specific competition behavior over the total number of observed occurrences of a specific competition type, averaged across all competitive pairs) for approach (mean ± SE: 45.3% ± 45.8%, *n* = 81), rope pulling (74.3% ± 33.8%, *n* = 33), freeloading (69.9% ± 43.4%, *n* = 63), monopoly (44.9% ± 44.7%, *n* = 16), and fight (20.8% ± 32.8%, *n* = 14). Elephants were least tolerant when partners displayed costly competition behaviors (monopoly and fight) toward them.

**Fig 3:**
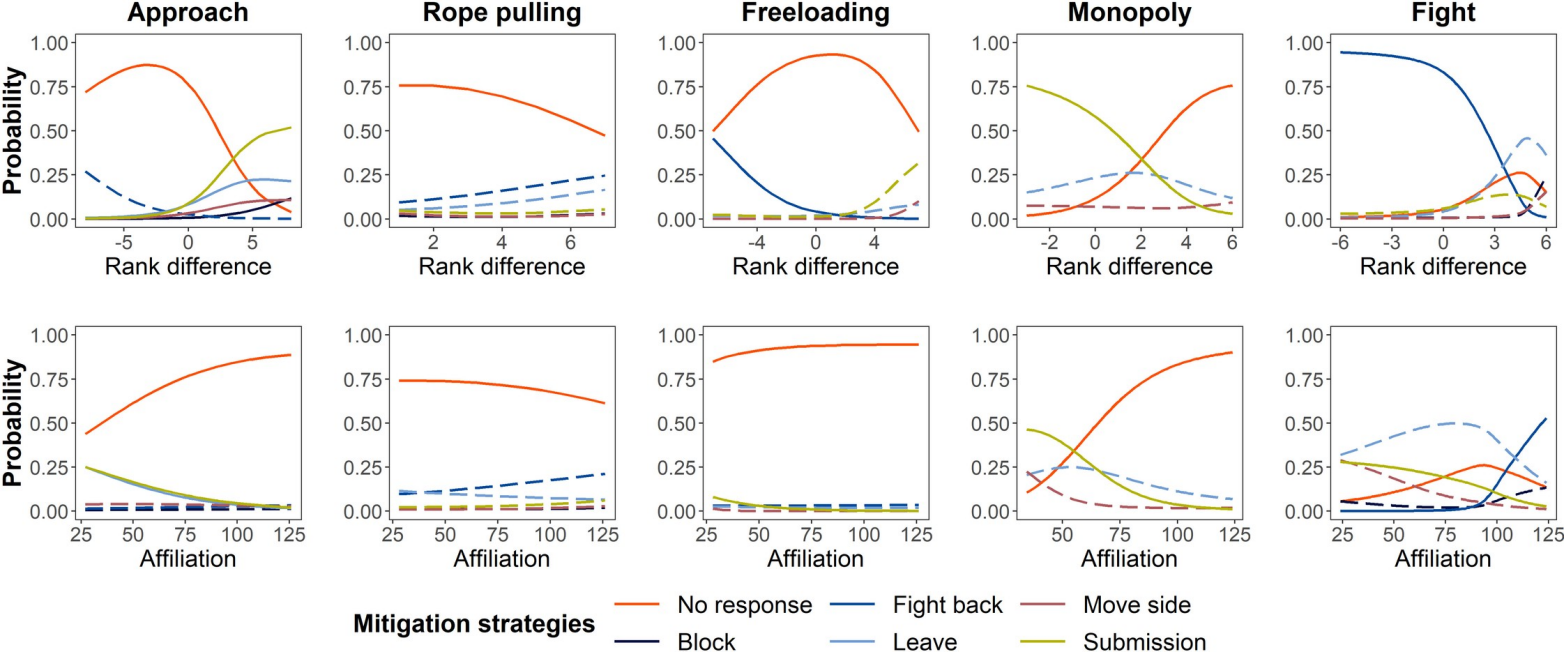
**Mitigation strategies used by elephants in Phase I based on their rank difference with a partner (top) or their level of affiliation (bottom).** Rank difference was calculated using the ranking of the initiator elephant minus the ranking of the target elephant. A positive rank difference means the initiator was dominant to the target elephant, while a negative value indicates the initiator was of lower rank. The only exception to this is that the rank difference under the “rope pulling” competition type was calculated as the absolute value of the rank difference between initiator elephant and target elephant, because we could not tell which individual initiated the behavior when 2 elephants pulled a single rope. The maximum affiliation index was 126. A solid line means that the probability of a mitigation strategy being used by an elephant can be predicted by rank difference (top plots) or affiliation (bottom plots), while long dashed lines represent nonsignificant patterns. The solid red line (no response) was set as a reference level in the models. The data used to generate this figure can be found in S1 Data.

Excluding “no response,” we recorded 5 types of mitigation strategies: submission, leave, block, fight back, and move side (see Table 2 for definitions and S4 Table for the frequency of each mitigation strategy). By fitting Bayesian multinomial regression mixed models, we tested if Asian elephants applied different strategies based on the affiliative closeness (index) and rank difference between the competitors under different competition scenarios. The occurrences of mitigation strategies in response to different types of competition are displayed in Fig 3, and the results of our between-elephant affiliation analysis (representing the closeness between elephants) are shown in S5 Table. Note that for Bayesian modeling results, we report the 95% credible intervals (CIs; reported within the text below and in S6 and S7 Tables). CI is reported as a range in brackets; if it does not cross zero, 0.00, the effect is significant.

**Table 2:** Mitigation strategies developed by elephants during the experiment and their definitions.

Mitigation strategy	Definition
Submission	Elephant pushes rump toward another elephant, to show their subordinate rank, in order to stay in the position near the rope.
Leave	Elephant leaves their position near the apparatus and quits cooperating.
Block	The elephant stands in front of the apparatus and uses their body to prevent another approaching elephant from coming near the apparatus.
Fight back	Elephant pushes or puts their trunk over another elephant (competition initiator) after a competition was initiated.
Move side	When an elephant approaches the target elephant standing in front of one end of the rope, the target elephant moves to the other rope end.

With the “approach” competition behavior, both rank difference and affiliation had significant effects on the mitigation strategies applied by the elephants. In response to the approach behavior, the target elephant showed more tolerance (no response) to those initiators that had a lower rank (for elephant rankings, see S1 Table). The probabilities of elephants using “block,” “leave,” “move side,” and “submission” as mitigation strategies were significantly different compared to “no response,” such that the probabilities increased as the rank of the competition initiator increased (Bayesian multinomial regression mixed model; for “block”: 95% CI: [0.10, 1.61]; for “leave”: 95% CI: [0.12, 0.97]; for “move side”: 95% CI: [0.15, 0.92]; for “submission”: 95% CI: [0.22, 1.26]; Fig 3; for more model details, see S6 Table). In one striking example, the elephant SMW used the strategy “move side” in response to the approach of a higher-ranking female (YMM); while SMW switched to the opposite side of the table, he pushed a higher-ranking elephant (PS) with whom he shared the highest affiliation toward YMM to displace her entirely in order to cooperate with PS (S5 Table, S2 Movie; this behavior was observed 15 times during the study). The probabilities of a target elephant using “submission” or “leave” strategies to avoid further competition compared to “no response” were significant, such that the probabilities increased as the affiliation index with the approaching elephant decreased (for “submission”: 95% CI: [−0.05, −0.02]; for “leave”: 95% CI: [−0.06, −0.02]; see Fig 3 and S6 Table). Elephants most often displayed the “approach” competition behavior with conspecifics of similar rank (S1A Fig).

Competition behavior while rope pulling usually occurred between highly affiliated elephants (S1B Fig). As mentioned above, the tolerance percentage (i.e., when elephants showed “no response”) was high in response to rope pulling. In an analysis of the rope-pulling behavior using a Bayesian multinomial regression mixed model, the probabilities of elephants using each mitigation strategy relative to showing “no response” were low and not significantly influenced by either the rank difference or affiliation index between elephants (for model details, see S6 Table; the pattern is illustrated in Fig 3).

Freeloading happened more frequently among elephants with similar ranks (S1A Fig). The difference between the probability of target elephants applying the “fight back” strategy compared to “no response” was statistically significant, such that the probability increased as the rank of the freeloader decreased (Bayesian multinomial regression mixed model; 95% CI: [−0.95, −0.18]). The probability of “submission” increased as the affiliation between the elephants decreased (95% CI: [−0.08, −0.02]). In response to freeloading, the probabilities of elephants applying the “leave” or “move side” strategies were low and not significantly influenced by rank difference or affiliation between elephants. The elephants did not use the “block” strategy at all (for model details, see S6 Table and Fig 3).

Because the trays were separate in Phase I and elephants usually ate at the same time, the frequency of the competitive behavior, “monopoly,” was low overall (frequency = 85 times, Fig 2A). When it occurred, high-ranking elephants were more likely to monopolize the food reward (S1A Fig). In response to monopoly behavior, elephants applied only the “leave,” “move side,” or “submission” strategies to avoid further competition. While the probabilities of the former 2 strategies were not affected by rank difference or affiliation, the probability of elephants applying the “submission” strategy was significantly different compared to “no response,” such that the use of “submission” decreased as the rank of the competition initiator increased (Bayesian multinomial regression mixed model; 95% CI: [−2.59, −0.24]) or as the affiliation with the initiator increased (95% CI: [−0.19, −0.04]; for model details, see S6 Table and Fig 3).

Finally, the competition behavior, “fight,” also happened at a relatively low frequency (frequency = 105 times, Fig 2A), and, usually, between elephants of different ranks and high affiliation (S1A and S1B Fig). Under this competition scenario, the probability of elephants fighting back was significantly different compared to “no response,” such that the probability increased as the rank of the initiator decreased (Bayesian multinomial regression mixed model: 95% CI: [−8.59, −0.36]) or as the affiliation with the initiator increased (95% CI: [0.03, 0.92]). The probability of the elephants using the “submission” strategy was significantly different compared to “no response,” such that the probability increased as the affiliation with the initiator decreased (95% CI: [−0.11, −0.00]). Elephants’ application of the “block,” “leave,” or “move side” strategies were not significantly influenced by rank difference or affiliation index (for more model details, see S6 Table and Fig 3).

### Phase II (1-tray condition): Competition

**Fig 4:**
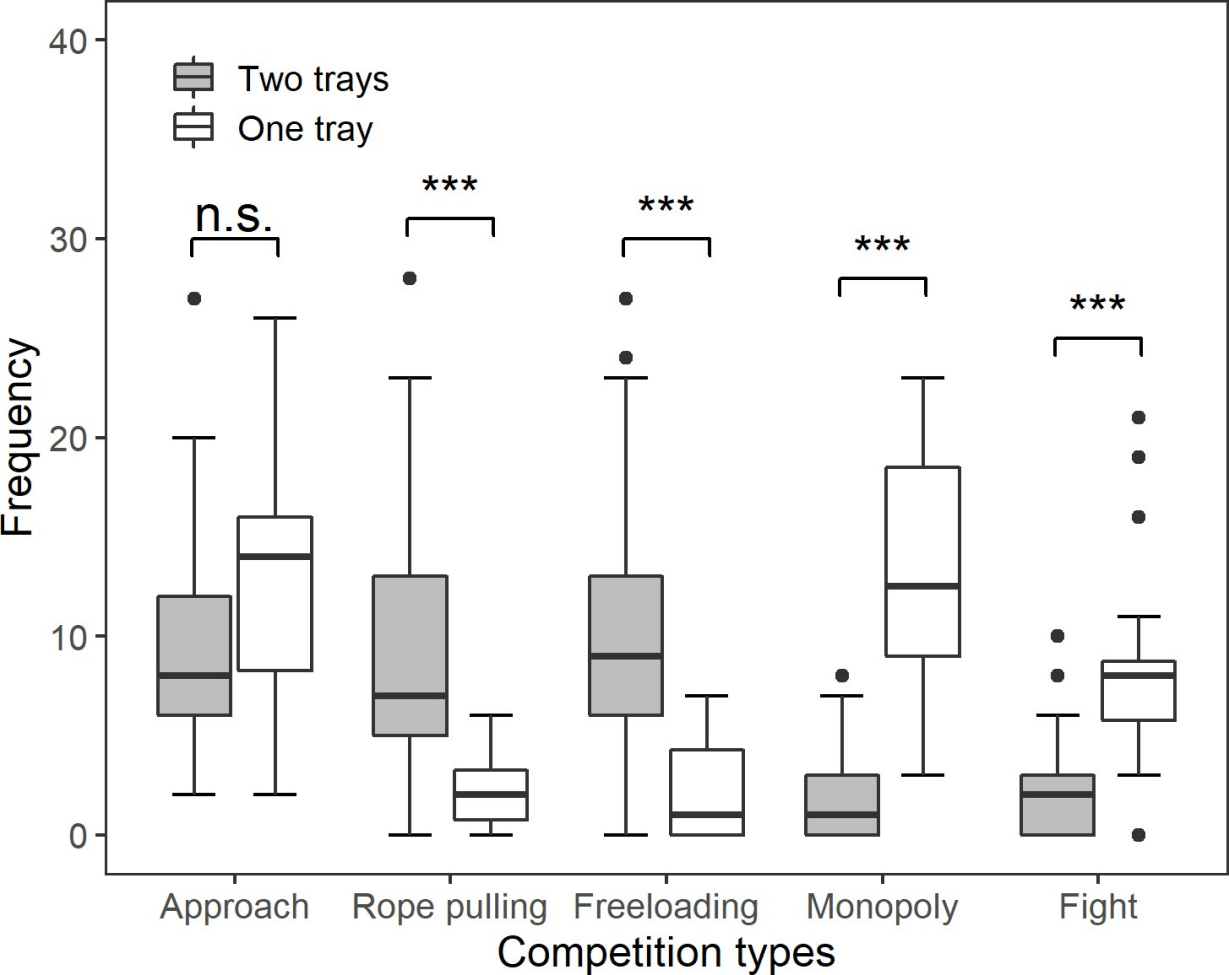
Comparison of competition behavior frequencies between 2-tray and 1-tray phases. The bold line inside each box is the median frequency per session of each competition behavior. The top whisker represents the maximum frequency, while the bottom whisker represents the minimum frequency (excluding outliers). Upper and lower quartiles are represented by the top and bottom edge of the box, respectively. The black data points above the top or below the bottom whisker are outliers. Asterisks represent significant differences between the frequency of behaviors between the 2-tray and 1-tray conditions (*** *P* < 0.001; n.s. *P* > 0.05). The data used to generate this figure can be found in S1 Data.

In Phase II, we placed only 1 tray in the center of the table, thus limiting the food resource and making it easier for a single elephant to monopolize it. This phase aimed to investigate whether cooperation between elephants would remain stable or break down under highly competitive circumstances. Welch *t* tests were used to investigate the difference in frequency of each competition type between Phase I (2-tray condition) and Phase II (1-tray condition). In the 1-tray condition, 2 very costly competition types, “monopoly” and “fight,” happened significantly more frequently than in the previous 2-tray condition (Fig 4; monopoly comparison: t = 7.521, df = 16.466, *P* < 0.001 and fight comparison: t = 4.293, df = 17.279, *P* < 0.001). Rope-pulling and freeloading behaviors, on the other hand, occurred significantly less frequently in the 1-tray than the 2-tray condition, while the frequency of approach behavior did not differ significantly between conditions (rope-pulling comparison: t = −6.210, df = 58.757, *P* < 0.001; freeloading comparison: t = −6.932, df = 58.697, *P* < 0.001; and approach comparison: t = 1.565, df = 20.661, *P* = 0.133). After 17 sessions of the 1-tray condition, cooperation broke down completely and never occurred again (Fig 1).

The mean comparisons of rank difference and affiliation under different competition types in the 1-tray phase are shown in S1C and S1D Fig, respectively. The change in competition behavior over time in the 1-tray phase is shown in Fig 2B.

### Phase II (1-tray condition): Mitigation strategies

**Fig 5:**
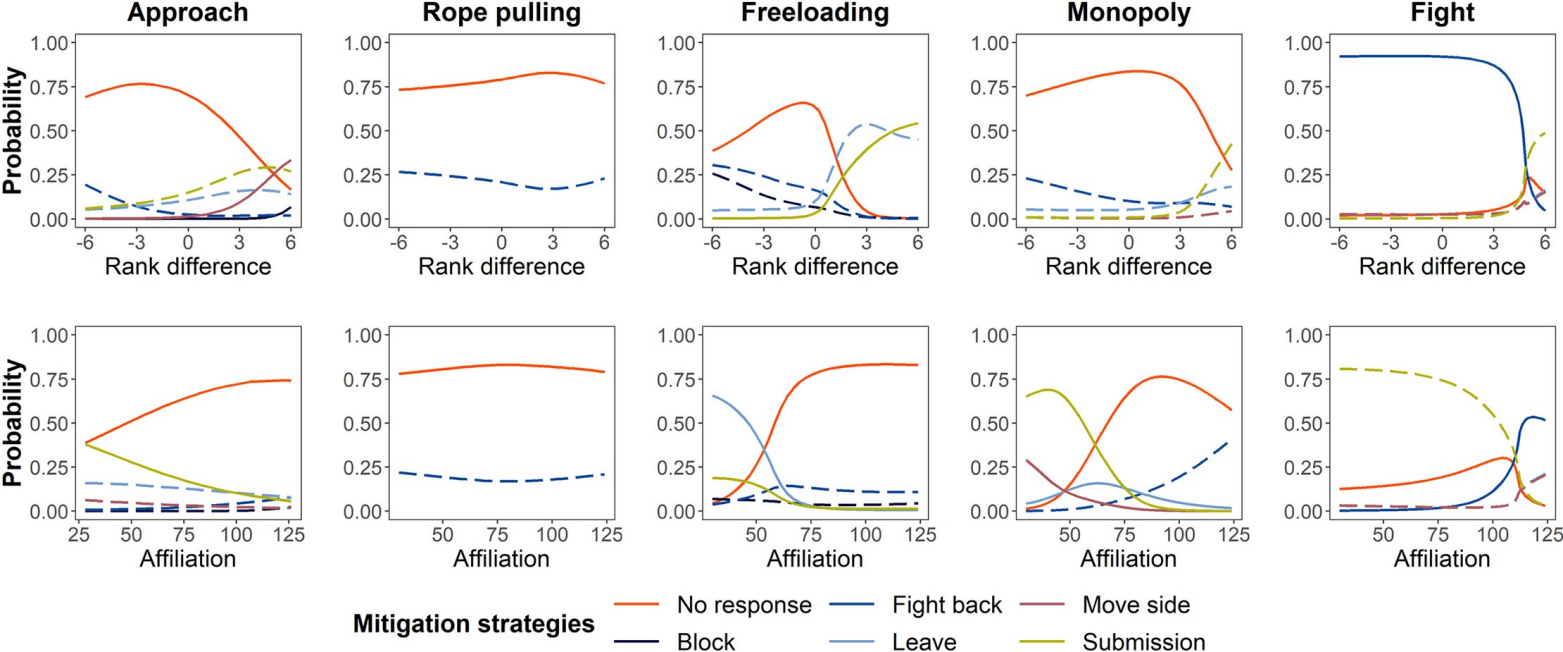
Mitigation strategies used by elephants in Phase II based on their rank difference with a partner (top) or their level of affiliation (bottom). Rank difference was calculated using the ranking of the initiator elephant minus the ranking of the target elephant. A positive rank difference means the initiator was dominant to the target elephant, while a negative value means the initiator was of lower rank. The only exception to this is that the rank difference under the “rope-pulling” competition type was calculated as the absolute value of the rank difference between the initiator elephant and the target elephant because we could not differentiate between the initiator and target when the 2 elephants were pulling a single rope. The maximum affiliation index was 126. A solid line means that the probability of a mitigation strategy being used by an elephant can be predicted by rank difference (top plots) or affiliation (bottom plots), while long dashed lines represent nonsignificant patterns. The solid red line (no response) was set as a reference level in the models. The data used to generate this figure can be found in S1 Data.

In this phase, in response to the “approach” competition behavior, the probabilities of target elephants using the “block” or “move side” strategies were significantly different compared to “no response,” such that the probabilities increased as the rank of the approaching elephant increased (Bayesian multinomial regression mixed model; for “block”: 95% CI: [0.08, 11.17]; for “move side”: 95% CI: [0.18, 2.23]). In addition, the probability of the target elephant submitting was significantly different than “no response,” such that as affiliation increased, the probability of submission decreased (95% CI: [−0.06, −0.01]). Other mitigation strategies were used in response to “approach,” but none were significantly influenced by rank difference or affiliation between elephants (for more model details, see S7 Table and Fig 5).

In response to rope pulling, elephants only used the “fight back” strategy, regardless of rank difference or affiliation index (Bayesian multinomial regression mixed model; for “rank difference”: 95% CI: [−1.78, 0.86]; for “affiliation”: 95% CI: [−0.11, 0.05]; for model details, see S7 Table; the pattern is illustrated in Fig 5).

When freeloading happened in this phase, the target elephants applied multiple strategies, excluding “move side.” The probability of elephants using “submission” was significantly different compared to “no response,” such that the probability increased as the rank of the initiating elephant increased (Bayesian multinomial regression mixed model; 95% CI: [0.79, 13.17]) or as the affiliation between the elephants decreased (95% CI: [−0.84, −0.00]). The probability of elephants leaving was significantly different compared to “no response,” such that the probability increased as the target elephant’s affiliation with the freeloader decreased (95% CI: [−1.00, −0.06]). They also applied the “block” and “fight back” strategies, but this application was not significantly influenced by rank difference or affiliation (for model details, see S7 Table and Fig 5).

In response to the “monopoly” competition behavior in this phase (S3 Movie), elephants applied the “fight back,” “leave,” “move side,” and “submission” strategies. Rank difference had no significant impact on the mitigation strategies elephants applied in response to “monopoly” as a competition behavior (for model details, see S7 Table and Fig 5). The probabilities of target elephants using “leave,” “move side,” or “submission” were significantly different compared to “no response,” such that the probabilities increased as the target elephant’s affiliation with the initiator decreased (Bayesian multinomial regression mixed model; for “leave”: 95% CI: [−0.15, −0.01]; for “move side”: 95% CI: [−0.74, −0.06]; for “submission”: 95% CI: [−0.45, −0.08]). Affiliation had no significant impact on the use of the “fight back” strategy (95% CI: [−0.01, 0.20]).

Finally, when elephants initiated “fight” competition behaviors, the probability of target elephants using the “fight back” mitigation strategy was significantly different compared to “no response,” such that the probability increased as the rank of the initiator decreased (Bayesian multinomial regression mixed model; 95% CI: [−125.72, −0.01]) or as their affiliation increased (95% CI: [0.02, 9.90]). Target elephants also applied “leave,” “move side,” or “submission” strategies in response to “fight”; however, the probabilities of their application were not significantly influenced by the rank difference or affiliation index between elephants (for model details, see S7 Table and Fig 5).

## Discussion

Our study, the first to investigate the varying levels of competition and cooperation in a controlled experimental study with elephants, showed that while elephants display competition behaviors frequently (the ratio of cooperation to competition was nearly 1:1), they maintain stable and effective cooperation by tolerating competition or using a variety of mitigation strategies to counter it. Similar patterns exist for primates [30], suggesting that comparable strategies for mitigating competition and maintaining cooperation may have evolved independently in evolutionarily distant species. In addition, when food resources were limited, costly competition behaviors (“monopoly” and “fight”) prevailed, leading to the breakdown of cooperation (Fig 1). This is consistent with what we know about human cooperation; monopolizing products generated by cooperative efforts can reduce the payoffs and jeopardize cooperation [14].

Our findings partly support the “emotional reactivity hypothesis” [18,19]—higher levels of tolerance allow for better performance during social problem-solving—as elephants displayed high levels of tolerance during the cooperation task. One reason this may be is that tolerating competition may act as a sort of compromise that still leads to a shared benefit. While low-cost competition behaviors may lead to a loss of position, temporary access to the rope, or a portion of the food reward, elephants may still obtain enough of the food reward for cooperation to be worthwhile. The competition behavior, “monopoly,” however, is highly costly relative to the others because it resulted in the partner elephant receiving no food reward. In addition, initiators of monopoly behaviors were usually elephants of higher rank (S1A Fig). Thus, elephants on the receiving end of such behaviors often submitted to (or showed no response and thus tolerated) the dominant individuals (Fig 3). Regardless of rank, once the benefits of cooperation no longer existed, cooperation broke down in our study.

It was clear the elephants wanted to continue cooperating and thus often adopted various strategies to mitigate competition. Among all the mitigation strategies elephants used, elephants seldom used “block” as a strategy, but when it was used, it was often directed at approaching elephants of higher rank. “Block” may not be an efficient strategy to defend against a higher-ranking elephant, hence its rarity. Instead, elephants often chose to be submissive toward a higher-ranking elephant or one with whom they shared a lower affiliation more often than toward one with whom they shared a higher affiliation, likely because the potential risk from a “nonfriend” was uncertain. Elephants applied the “submission” strategy in all competition scenarios recorded in our study. This makes sense because being “submissive” might allow elephants to stay close to the food reward without arousing further competition. However, when elephants of a lower rank or with a higher affiliation index initiated a “fight” or “freeloading” competition behavior, target elephants would often use a “fight back” strategy to directly protest the competition behavior, likely because elephants were unwilling to tolerate such competition from those either below them in rank or closely affiliated with them (Fig 3). Interestingly, “freeloading” happened frequently between elephants with similar rank (S1A and S1C Fig), and target elephants in these pairings rarely fought back against it. This may be because elephants of similar rank tolerate each other’s behavior more to avoid the risk of losing rank status in a fight.

Furthermore, the probability of elephants choosing to “leave” was the highest when the 2 costly competition behaviors “monopoly” and “fight” happened in 2-tray Phase I. Although it was not significantly affected by rank difference or affiliation, overall, elephants chose to quit cooperation when costly competition behaviors occurred. The varying impact of rank difference and affiliation on the use of particular competition behaviors and the mitigation strategies employed in response to them suggests that elephants understand each other’s role and status in cooperation and behave flexibly in competitive interactions.

One interesting mitigation strategy that was sometimes employed was “move side.” In this scenario, when elephants chose not to tolerate a competitive individual (i.e., when they stopped showing “no response”), they might have responded by moving to another side to avoid further competition. Interestingly, when SMW, an elephant highly affiliated with the most dominant elephant in the group (named PS), was approached by a third party (named YMM), he applied the “move side” strategy and would gently push PS to the side he was originally on to exclude YMM (S2 Movie). SMW would then take the rope farthest from YMM and cooperate with PS. While we acknowledge there may be simpler, more parsimonious explanations for this behavior, one possibility is that SMW may have used a strategy that allowed him to exploit his relationship with the dominant elephant to maximize his opportunities for cooperation. By manipulating a dominant conspecific to prevent a third party from impacting his own opportunity to cooperate, SMW may have reduced the cost brought by potential competition and maximized his own payoffs. Another example of using third-party interactions to impact cooperation involved the female, NAA, who was of higher rank than SMW. She would often stand close to SMW, who had a higher affiliation with a more dominant male elephant than she did; this allowed her to gain access to the rope over SMW or to share the food reward with him when the dominant pulled the other end of the rope. These behaviors support the idea that elephants may understand relationships between other elephants and use this information to maximize the benefits of cooperation [63,64].

Consistent with research on capuchin monkeys and humans [14,65], when a dominant elephant monopolized the food over a subordinate in the Phase II 1-tray condition, which could potentially lead to a fight between them, cooperation suffered a dramatic and rapid drop. We observed that it was always the subordinate elephant that lost interest and left the cooperation task first; dominant individuals never refused to cooperate in Phase II. This indicates that cooperation broke down when subordinate individuals chose to abandon cooperation efforts that led to little or no food reward. It is possible this result could be based on reciprocity or the lack thereof and future expectations for food rewards. If monopolization of food happens frequently in cooperative tasks, subordinate individuals may have no expectation of a future benefit and thus may abandon cooperation [1,66].

Unlike in humans [14,67], who share food when cooperation is maintained long term even under intense within-group competition, elephants never actively shared food in our test. Self-interest in the form of food monopolization led to abandonment of the task in our elephant group, resulting in a total breakdown in cooperation [68,69]. Given what we know about elephant foraging behavior and sociality, however, these results are not surprising. While some nonhuman primate species hunt scarce resources and thus have opportunities in the wild to monopolize food or compete for access to it [62,70–73], Asian elephants are generalist feeders, browsing and grazing on a variety of vegetation. Because of this, opportunities for monopolizing food in the wild are extremely limited. Thus, while our study illustrates how cooperation can be maintained and subsequently extinguished in an experimental task and thus suggests that the underlying mechanisms for cooperative behavior may be, at least in part, analogous across species, our paradigm may lack ecological validity for elephants. The complex sociality known to exist in elephants may instead be driven by the need for cooperative care of young or predator defense, rather than any balance of food resource sharing. These cooperative relationships may also be affected by the fact that rank differences within related family groups in the wild may differ from the rank order that develops within a semi-wild or captive population. Future studies that look at the mitigation of competition in elephants in nonfood sharing contexts and within both kin and nonkin groups might contribute substantially to our understanding of the evolution of cooperation across species.

In conclusion, our study indicates that elephants employ a number of mitigation strategies to maintain cooperation even in the face of diverse competitive behaviors. Similarities in the expression of complex cooperative tendencies across evolutionarily distant species support the idea of convergent cognitive evolution (i.e., the independent evolution of similarities in cognition likely due to similar selection pressures on behavior rather than common ancestry). This makes sense in light of what we are learning about elephant cognition, particularly in terms of their sociocognitive complexity (e.g., partner coordination [60], empathy [45], and consolation [44]). Like humans and other primates, elephants work with partners and mitigate competition based on relationship quality and social ranking and prevent or avoid conflict with third parties, suggesting a capacity for behavioral flexibility when faced with tasks requiring social problem-solving. This study supports the need for further research on elephant social behavior and cognition, as well as the idea that, perhaps counterintuitively, a better understanding of the evolution of human sociality may come from studies focused not solely on the primate taxa but also on other big-brained nonprimates as well.

## Methods

### Subjects

Nine semi-wild Asian elephants from the MHW Elephant Camp in Taikkyi, Yangon, Myanmar, all owned by the MTE, were included in this study. This sample of 9 elephants (F = 5, M = 4) ranged in age from 6 to 55 years old (see S1 Table for elephant demographics). All of the elephants were trained at the age of 5 to follow simple commands such as “go,” “stop,” “come,” etc. The MTE has now begun to integrate positive reinforcement training with traditional methods [41], specifically as a result of the recent end to commercial logging in 2016. The elephants in the MTE camps that participated in the current study were “retired” from work and resided in the MTE camps full time. The cost of their care was covered in full by the government. Each elephant had 1 elephant handler (“mahout” or “oozie”) whose job is exclusively to care for the elephant to whom they are assigned. The mahout is usually assigned for indefinite periods of time to a single elephant. Every morning, mahouts collected their elephants from the forest to check their health and bathe them before releasing them back to the surrounding forest. Elephants were also given regular veterinary care as needed by the MTE staff veterinarians. Comparable to some other captive elephant populations in Southeast Asia, the MTE elephants living in the MHW camp in this study live a semi-wild lifestyle with regular access to forests to roam free. The introduction of our cooperation apparatus allowed for both behavioral and cognitive enrichment on a completely voluntary basis.

Approximately 20 wild elephants also lived in the area. The semi-wild MTE elephants in this landscape interact with the wild elephants, and there is interbreeding between the groups (the first author, LL, observed these interactions near the elephant camp). Because the semi-wild elephants have significant opportunities to interact or cooperate with other elephants when they are in the forest, but have been trained extensively to respond to human cues, they make ideal participants for a study of cooperation in a controlled environment.

We first selected 10 elephants from the semi-wild group, with the following criteria: (1) they had previously been trained to pull ropes (as working elephants, they were trained to pull ropes during logging activities); (2) they responded well when their mahouts went to collect them from the forest each day; (3) they had no history of violence toward humans (this criterion was used to ensure the safety of the mahouts and the primary experimenter, LL); and (4) the mahouts were comfortable safely testing these elephants together. None of the female elephants were pregnant at the time of testing nor did they need to be separated from their original social groups. One of the elephants did not pass apparatus training (see the “Experimental procedure” section below) and thus was excluded from testing.

### Ethics statement

This study was reviewed and approved by the MTE, protocol No.4527/MTE/AA(K)18. Our study obtained animal ethics approval (No. XTBG-2020-10) from the Xishuangbanna Tropical Botanical Garden.

### Apparatus setup

**Fig 6:**
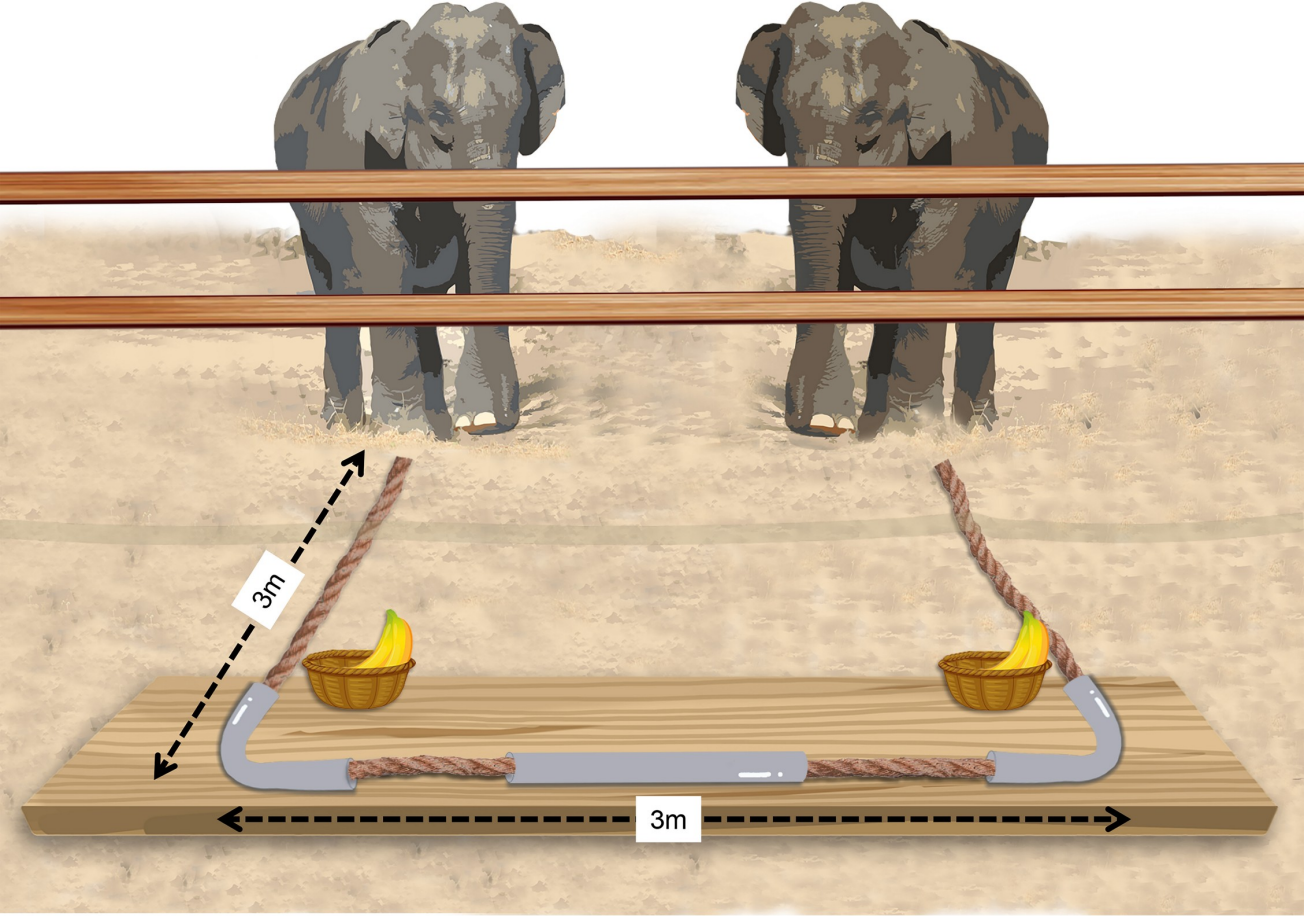
The loose-string cooperation apparatus in this study.

In a classical cooperation study from the 1930s, an out-of-reach platform had counterweights (i.e., it was heavy) that required the joint effort of 2 chimpanzees to retrieve an out-of-reach food reward [74]. To avoid the need to apply a counterweight heavy enough so that 2, but not 1, elephants could pull the platform, we adopted the loose-string apparatus developed by Hirata and Fuwa [54] for chimpanzees and adapted by Plotnik and colleagues [60] for elephant cooperation (Fig 6). A single rope was threaded through, behind and around a table, so that it could only be brought in when both ends of the rope were pulled. When only one side of the rope was pulled without the other, the rope became unthreaded from the now immovable table. Two trays were placed equidistant apart on either end of the out-of-reach table and baited randomly with 2 pieces of bananas or tamarind balls as food rewards. When and if both elephants pulled the rope at the same time, the food trays became accessible as the table moved toward them. The distance between the 2 rope ends was 3 m; thus, it was not possible for one elephant alone to pull both of them simultaneously.

We built a 38 × 38 m^2^ enclosure on flat grassland near the elephant camp as the testing arena. The apparatus was placed outside of the enclosure so that the table was not accessible to the elephants at the beginning of a trial (Fig 6). We set 3 video cameras (SONY (Tokyo, Japan) HDR-PJ760) to record their behaviors from the front, side, and back views of the enclosure.

### Experimental procedure

#### Training

Initial training aimed to ensure that elephants were sufficiently motivated to pull the rope ends to retrieve the food reward. We first attached a rope to the front of the table, so that an elephant could pull in the table with a single rope (see Plotnik and colleagues [60] for similar training on which the current procedure is based). A tray was placed on the table at its center and baited with 2 bananas. Each elephant was introduced to the location at the end of the rope in the absence of all other elephants. The elephant was free to pull the rope at will. If the elephant was unmotivated to pull the rope for 30 seconds, the mahout asked the elephant to pull. When the table was pulled in, if the elephant did not take the bananas from the tray, the first author (LL) guided the elephant to take the bananas; LL picked them up and dropped them back on the tray to draw the elephant’s attention with auditory cues. The process was repeated until the elephant pulled the rope without being prompted by the mahout 6 consecutive times.

We then threaded the rope through and around the table and trained pairs of elephants to pull the rope ends jointly to obtain food rewards. Using a random number generator, we paired 10 elephants randomly (*N* = 5 pairs). Elephants were introduced into the enclosure in front of the apparatus, pair by pair, in the absence of other elephants. While each mahout rode on the elephant’s neck (the easiest way to give commands to the elephant during training), they told their respective elephants to pull each rope together, at the same time. If an elephant pair successfully pulled the rope ends together (i.e., if the pairs pulled in the apparatus and retrieved the food rewards together), mahouts then got down from the elephants and provided commands from the ground. When the elephants pulled together successfully in 3 consecutive trials, the mahouts left the area, and elephants were free to pull the rope by themselves. Training was complete when elephant pairs successfully retrieved the food rewards by pulling in the table on their own 6 times. If elephants failed to reach this criterion after 60 unsuccessful attempts (or, if an elephant was unwilling to pull the rope with a partner and walked away), we stopped their training and reassigned the elephants to another individual, and the training process was repeated for the new pair on the next day.

On the next day following successful training, we tested the elephant pairs again without the mahouts present in three 10 trial sessions (only 1 session was run per day over 3 days). We rebaited the food trays after each trial. If the elephants did not successfully pull in the apparatus after 1 minute or only 1 elephant pulled the rope causing it to become unthreaded, the trial was marked as a failure. Elephants were included in testing if they succeeded in at least 5 out of 10 trials in at least 2 of 3 sessions. Elephants that did not reach the criterion (due to loss of interest or they failed to pull) were to be excluded from testing. One elephant (SKL) lost interest and thus failed to reach criterion, while her partner (NS) was included in testing due to her success when paired with another elephant.

#### Testing

Testing in Phase I explored whether elephants would maintain cooperation over time, and, if there was any competition during interactions with the apparatus, how elephants would mitigate it. Testing was conducted either in the early morning or late afternoon. Each session lasted 1 hour. First, 9 elephants were introduced into the enclosure and allowed to freely interact with one another. Researchers and mahouts remained outside of the enclosure for the duration of a session. Two mahout leaders manipulated/reset the apparatus as needed, and 2 researchers recorded the data. Both trays were baited randomly with 2 pieces of banana or 2 tamarind balls at the beginning of each trial, and both ends of the rope were made accessible to the elephants. When the elephants successfully cooperated and obtained the food rewards, researcher A (a research assistant from Myanmar, Zin Nwe Soe) marked down S (success) and recorded the name of the elephants that pulled the rope and ate the food rewards. After 1 minute (the intertrial interval), trays were baited again, the 2 mahout leaders reset the apparatus, and a new trial began. If the elephants failed to cooperate—if one elephant pulled one end of the rope without waiting for another elephant and the other end of rope became inaccessible—researcher A marked down F (failure) and recorded the name of the elephant that pulled the rope. If elephants showed no interest in pulling the rope for 5 minutes, a researcher also marked the trial as a failure. Within one 1-hour session, elephants could participate in as many trials as time allowed. We conducted 1 session every 2 days to maintain the elephants’ motivation to participate. In Phase I, we conducted 45 sessions in total (45 days). The second researcher, B (the first author, LL), was responsible for recording competition behaviors and any subsequent mitigation strategies throughout the experiment. If elephants showed “no response” when competitors initiated any type of competition, this was identified as “tolerance.” Physical proximity was not used as a measure of tolerance as this was more an indicator of social closeness or affiliation (see the following “Affiliation index and dominance ranking” section).

#### Enhanced competition

In testing Phase II, a single, central tray with food was placed in the middle of the table to explore whether elephants would still maintain cooperation under a condition that might promote competition (i.e., when food rewards could be monopolized). One session lasted for 1 hour. After each trial, we waited 1 minute before resetting the apparatus, as we did in Phase I. The tray was baited randomly with 2 bananas or 2 tamarind balls. In Phase II, we conducted 23 sessions in total (23 days).

We were aware that introducing a competitive component to the cooperation tests might increase the chances of aggressive behavior. We also recognized that any social cognition task with elephants that involved multiple interactions between unrelated individuals could potentially lead to competitive interactions that could escalate into dangerous aggression. The mahouts had extensive experience working with this population of elephants and informed our selection of elephants for this study based on their existing relationships. During testing, mahouts remained near the enclosure for safety reasons, but were instructed to remain out of sight of the elephants and only to vocalize toward the elephants to break up severe fighting if and when it occurred. The experimental area was large enough that it allowed elephants to escape from the perimeter around the table if necessary. In fact, there was only one instance that required the mahouts’ intervention throughout the course of this study; YMM once chased SMW within the enclosure, which led to SMW escaping through the fence. Both elephants were returned to the enclosure, and the aggression was not repeated. In general, mahouts and experimenters agreed that a trial would be stopped and the elephants removed from the testing area if any potentially dangerous, highly aggressive interactions were observed. If at any point the mahouts had believed there was an increased risk of injury to human or elephant, the experiment would have been halted immediately. In addition, the entire study was voluntary on the elephants’ part; if elephants refused to participate (they never did in this study but have in other studies we have conducted with elephants), they would be excluded from cooperation trials. Elephants are highly social animals where conflict is relatively rare [44]; we provided the elephants with opportunities to refuse to cooperate and to avoid conflict if they so chose, limiting chances for any overly aggressive interactions during the study or following its completion. In fact, mahouts reported no negative changes to the elephants’ relationships nor any increase in aggressive behavior between elephants after the study was completed, suggesting this experiment had no unintended negative consequences on the elephants’ social behavior.

### Affiliation index and dominance ranking

Elephants often stand in close proximity to family members in their social groups [42]. Although the elephants in our sample were not in natural, related family groups, they did form relationships with each other over time. Thus, to measure the closeness/relationship of each dyadic pair in our experiments, we used the frequency of neighbors recorded as an affiliation index. Every 4 days, mahouts collected the elephants together in a yard at around 7:00 AM, and researchers observed the elephants from a 2-m high watchtower in the yard. Using scan sampling, we recorded when elephants were within 5 meters of each other [75]. We scanned 3 times with 10-minute intervals between each scan sample and thus had 3 scans per observation day. Thus, over a 44-day period, we had a total of 132 scans for proximity.

While researcher A recorded proximity using scan sampling, researcher B used all-occurrence sampling to record dominance interactions between elephants (see S8 Table for definitions), including the interaction type, which elephant was the initiator, and which elephant was the recipient/target. Data were collected for 30 minutes each day for 44 days, resulting in a total of 1,320 minutes of observation. We then used David’s score to calculate the dominance ranking of each elephant [76].

### Statistical analyses

We used a Spearman correlation analysis to explore if cooperation success rate significantly increased or decreased with time in both the 2-tray and 1-tray testing phases (Phase I and II, respectively). By applying generalized additive models in R package “mgcv” [77], we fitted the frequency of competition behaviors of each type as the response variable and session number as an independent variable to test if the pattern of competition behaviors was significant in both the 2-tray and 1-tray phases. To compare rank difference and the affiliation index of elephant dyads between different competition behaviors, we applied a hierarchical cluster algorithm using R package “ScottKnott” [78].

Within the 2-tray phase, with each competition type, we calculated the tolerance levels by proportion of “no response” in all mitigation strategies. To explore the relationship between rank difference and mitigation strategies applied under each competition type, and how affiliation influenced the frequency of strategies used, we fit Bayesian multinomial regression mixed models using R packages “brms” [79] and “rstan” [80]. Bayesian multinomial regression mixed modeling allows for the fitting of categorical response variables and for the inclusion of random effects such as elephant ID [81] and promotes the convergence of models when sample size is relatively small. In the Bayesian models, we set rank difference and affiliation as independent variables, mitigation strategies as the response variable, and the ID of competition initiators and recipients as random effects. These models were fit with noninformative priors. We set “no response” as the reference level and estimated the probability of the rest of the mitigation strategies relative to it. In the models, we set up 4 chains and 2,000 iterations, removed the results of the first half of iterations, and increased the times for iteration if the sample size was not enough to achieve model convergence (the raw data and R code are included in S1 and S2 Data, respectively). We also explored the relationship between both rank difference and affiliation and the mitigation strategies applied under each competition type in the 1-tray phase using the same statistical methods. Finally, Welch *t* tests were used to investigate the difference in frequency of each competition type between the 2-tray cooperation phase (I) and the 1-tray competition phase (II).

Two researchers recorded the success and failure to cooperate (researcher A), types and frequency of competition, and the elephants’ responses to competition (i.e., mitigation) during the experiment (researcher B). These data were recorded live. All sessions were also video recorded. An independent video coder who was blind to the design and hypotheses of the study coded 20% of the sessions chosen randomly from all of the videos. Interrater reliability [82] was high using Cohen κ for cooperation success: κ = 0.98; competition behaviors: κ = 0.96; and responses of elephants: κ = 0.98 (see S3 Data for data used to calculate interrater reliability). All statistical analyses were done in R 3.6.3 [83].

## Supporting information

S1 Table Demographics of semi-wild Asian elephants in the study, with their ranking. (PDF)

S2 Table Performance of elephants in training phase. (PDF)

S3 Table Contribution by and reward for each elephant in 2-tray Phase I and 1-tray Phase II. (PDF)

S4 Table Frequency of each mitigation strategy in 2-tray Phase I and 1-tray Phase II. (PDF)

S5 Table Affiliation index between elephants. (PDF)

S6 Table The model results on the impacts of rank difference and affiliation on the mitigation strategies selected by elephants in the 2-tray Phase I. (PDF)

S7 Table The model results on the impacts of rank difference and affiliation on the mitigation strategies selected in the 1-tray Phase II. (PDF)

S8 Table Dominant behaviors of elephants and behavior definitions. (PDF)

S1 Fig Means of rank difference and affiliation by competition type in 2-tray Phase I and 1-tray Phase II. **(A)** Means of rank difference by competition type in 2-tray Phase I. **(B)** Means of affiliation by competition type in 2-tray Phase I. **(C)** Means of rank difference by competition type in 1-tray Phase II. **(D)** Means of affiliation by competition type in 1-tray Phase II. The points represent the mean values, while lines are the range of the rank difference or affiliation. Different colors represent a significant difference between behaviors, while the same color (blue) represents a nonsignificant difference. Rope pulling was not included in the means comparison of rank difference because it used a different calculation for rank difference (see Figs 3 or 5 for details). The data used to generate this figure can be found in S1 Data.
(TIFF)

S1 Movie Cooperation between elephants in 2-tray Phase I. Two elephants (PS on the left, SMW on the right) pull the rope simultaneously to obtain the food in the food tray.
(MP4)

S2 Movie “Move side” competition strategy in 2-tray Phase I. When a higher-ranking female elephant (YMM) stood toward one side of the table, a lower-ranking male elephant (SMW) at the opposite side of the table pushed his dominant partner (PS) toward the side at which the female (YMM) was standing (see approximately 00:43 in the clip). Since SMW’s partner, PS, was the most dominant, they were able to cooperate and exclude the female from the cooperative task. At the end of the trial, SMW tolerated NAA, a female, sharing from his tray.
(MP4)

S3 Movie Cooperation between elephants in 1-tray Phase II and the monopoly competition behavior. The dominant elephant (PS, on the left side in the video) took all the food in the tray after cooperating with the other, lower-ranking female (YMM).
(MP4)

S1 Data Combined raw data. This Excel document presents the raw data, separated by worksheet/tab, for Figs 1–5 and the Supporting information figure (S1 Fig).
(XLSX)

S2 Data RAR file with R code. This file contains the R code used for statistical analyses and data plotting.
(RAR)

S3 Data Interrater reliability raw data. This Excel document presents the raw data used to calculate interrater reliability.
(XLSX)

## Abbreviations

CI: credible interval
MHW: Myaing Hay Wun
MTE: Myanma Timber Enterprise
