# Supplementary material for: Cooperating elephants mitigate competition until the stakes get too high
Source: PLoS Biol. 2021 Sep 28;19(9):e3001391. doi: 10.1371/journal.pbio.3001391 (PMC8478180; doi:10.1371/journal.pbio.3001391)
Supplement: S1 Table — (PDF) [file pbio.3001391.s001.pdf]

S1 Table. Demographics of semi-wild Asian elephants in the study, with their ranking.

No.	Name	Age (years)	Sex	Rank
1	SMW	8	Male	3
2	HLM	10	Male	2
3	WZS	12	Male	8
4	PS	20	Male	9
5	NAA	11	Female	4
6	NS	11	Female	5
7	NHH	6	Female	1
8	YMM	22	Female	6
9	KSK	55	Female	7
10	SKL	36	Female	NA*

*SKL did not pass the training phase, thus she was excluded in the ranking calculation.
