# Supplementary material for: Cooperating elephants mitigate competition until the stakes get too high
Source: PLoS Biol. 2021 Sep 28;19(9):e3001391. doi: 10.1371/journal.pbio.3001391 (PMC8478180; doi:10.1371/journal.pbio.3001391)
Supplement: S2 Table — (PDF) [file pbio.3001391.s002.pdf]

S2 Table. Performance of elephants in training phase.

Training phase	Elephant	Trials	Success in last 6 trials
1) Pull by single elephant	SMW	7	Pass
	HLM	6	Pass
	WZS	7	Pass
	PS	12	Pass
	NAA	7	Pass
	NS	7	Pass
	NHH	7	Pass
	YMM	7	Pass
	KSK	7	Pass
	SKL	6	Pass
2) Pull by elephant pair	YMM-NAA	30	Fail
	NS-NAA	71	Pass
	KSK-SKL	62	Fail
	HLM-SMW	62	Pass
	WZS-NHH	60	Fail
	NS-SKL	36	Pass
	YMM-PS	24	Pass
	KSK-HLM	50	Pass
	NHH-NAA	40	Pass
	WZS-SMW	39	Pass
3) Test	NS-NAA	30	Test 1(Pass)-2(Pass)-3(Pass)
	HLM-SMW	30	Test 1(Pass)-2(Pass)-3(Pass)
	NS-SKL	30	Test 1(Fail)-2(Fail)-3(Fail)

	YMM-PS	30	Test 1(Pass)-2(Pass)-3(Fail)
	KSK-HLM	30	Test 1(Pass)-2(Pass)-3(Pass)
	NHH-NAA	30	Test 1(Pass)-2(Fail)-3(Pass)
	WZS-SMW	30	Test 1(Pass)-2(Pass)-3(Pass)
