# Supplementary material for: Cooperating elephants mitigate competition until the stakes get too high
Source: PLoS Biol. 2021 Sep 28;19(9):e3001391. doi: 10.1371/journal.pbio.3001391 (PMC8478180; doi:10.1371/journal.pbio.3001391)
Supplement: S3 Table — (PDF) [file pbio.3001391.s003.pdf]

**S3 Table. Contribution by and reward for each elephant in two-tray phase I and one-tray phase II.** 'Contribution' represents the total frequency of rope-end pulls by each elephant. The 'reward' number outside the parentheses represents the total number of times the elephant retrieved food from a tray (i.e., the total number of times the elephant retrieved food from its own tray, the other elephant's tray or the shared tray). The first number in parentheses represents the number of times the elephant "freeloaded' food from a tray, while the latter number represents the frequency of 'monopolized' trays.

| Two-tray phase I | | | One-tray phase II | | |
|---|---|---|---|---|---|
| Elephant | Contribution | Reward | Elephant | Contribution | Reward |
| PS | 1423 | 1438 (63, 83) | PS | 423 | 302 (21, 190) |
| SMW | 991 | 740 (51, 0) | SMW | 295 | 119 (3, 16) |
| YMM | 777 | 637 (15, 1) | YMM | 165 | 9 (2, 5) |
| NAA | 200 | 210 (88, 1) | NAA | 101 | 18 (12, 2) |
| KSK | 187 | 78 (5, 0) | KSK | 24 | 4 (0, 2) |
| NS | 89 | 67 (9, 0) | NS | 7 | 0 (0, 0) |
| HLM | 46 | 25 (3, 0) | HLM | 1 | 0 (0, 0) |
| WZS | 16 | 13 (2, 0) | WZS | 3 | 0 (0, 0) |
| NHH | 9 | 0 (0, 0) | NHH | 2 | 0 (0, 0) |
