# Supplementary material for: Cooperating elephants mitigate competition until the stakes get too high
Source: PLoS Biol. 2021 Sep 28;19(9):e3001391. doi: 10.1371/journal.pbio.3001391 (PMC8478180; doi:10.1371/journal.pbio.3001391)
Supplement: S4 Table — (PDF) [file pbio.3001391.s004.pdf]

S4 Table. Frequency of each mitigation strategy in two-tray phase I and one-tray phase II.

Two-tray phase I					
Mitigation strategy	Approach	Rope pulling	Freeloading	Monopoly	Fight
No response	219	280	384	26	54
Submission	95	14	35	47	22
Block	15	2	0	0	1
Move side	42	2	3	5	6
Fight back	25	97	38	0	6
Leave	36	12	12	7	16
Total	432	407	472	85	105
**One-tray phase II**					
Mitigation strategy	Approach	Rope pulling	Freeloading	Monopoly	Fight
No response	76	29	17	133	45
Submission	49	0	4	29	30
Block	1	0	1	0	0
Move side	31	0	0	5	3
Fight back	9	6	4	23	55
Leave	36	0	8	25	5
Total	202	35	34	215	138
