# Supplementary material for: Cooperating elephants mitigate competition until the stakes get too high
Source: PLoS Biol. 2021 Sep 28;19(9):e3001391. doi: 10.1371/journal.pbio.3001391 (PMC8478180; doi:10.1371/journal.pbio.3001391)
Supplement: S5 Table — (PDF) [file pbio.3001391.s005.pdf]

S5 Table. Affiliation index between elephants. Affiliation index was calculated based on proximity data (i.e., the frequency of close affiliations between individuals) collected during instantaneous scan sampling to measure the relationship of each dyadic pair in our experiment.

No.	Elephant1	Elephant2	Affiliation Index	No.	Elephant1	Elephant2	Affiliation Index
1	PS	SMW	124	19	YMM	HLM	56
2	PS	YMM	50	20	YMM	WZS	24
3	PS	NAA	57	21	YMM	NHH	92
4	PS	KSK	41	22	NAA	KSK	59
5	PS	NS	65	23	NAA	NS	126
6	PS	HLM	74	24	NAA	HLM	37
7	PS	WZS	30	25	NAA	WZS	34
8	PS	NHH	79	26	NAA	NHH	96
9	SMW	YMM	30	27	KSK	NS	69
10	SMW	NAA	39	28	KSK	HLM	39
11	SMW	KSK	28	29	KSK	WZS	10
12	SMW	NS	41	30	KSK	NHH	54
13	SMW	HLM	89	31	NS	HLM	50
14	SMW	WZS	76	32	NS	WZS	28
15	SMW	NHH	65	33	NS	NHH	117
16	YMM	NAA	106	34	HLM	WZS	35
17	YMM	KSK	50	35	HLM	NHH	50
18	YMM	NS	121	36	WZS	NHH	30
