# Supplementary material for: Cooperating elephants mitigate competition until the stakes get too high
Source: PLoS Biol. 2021 Sep 28;19(9):e3001391. doi: 10.1371/journal.pbio.3001391 (PMC8478180; doi:10.1371/journal.pbio.3001391)
Supplement: S6 Table — (PDF) [file pbio.3001391.s006.pdf]

S6 Table. The model results on the impacts of rank difference and affiliation on the mitigation strategies selected by elephants in the two-tray phase I. 95% CI represents 95% credible intervals.

**Model** (competitive behavior)	**n**	**Predictor**	**Response**	**Estimate**	**Std. error**	**Lower 95% CI**	**Upper 95% CI**
**Approach**	432	Rank difference	No response*				
			Block	0.72	0.38	0.10	1.61
			Fight back	-0.32	0.23	-0.81	0.10
			Leave	0.50	0.21	0.12	0.97
			Move side	0.51	0.20	0.15	0.92
			Submission	0.69	0.26	0.22	1.26
		Affiliation	No response*				
			Block	-0.00	0.02	-0.04	0.03
			Fight back	0.00	0.01	-0.02	0.02
			Leave	-0.04	0.01	-0.06	-0.02
			Move side	-0.01	0.01	-0.03	0.00
			Submission	-0.04	0.01	-0.05	-0.02
**Rope pulling**	407	Rank difference	No response*				
			Block	0.12	0.71	-1.26	1.57
			Fight back	0.28	0.26	-0.24	0.78
			Leave	0.36	0.31	-0.22	1.04
			Move side	-0.21	0.89	-2.05	1.55
			Submission	0.05	0.67	-1.16	1.45
		Affiliation	No response*				
			Block	0.01	0.03	-0.05	0.07
			Fight back	0.01	0.01	-0.01	0.03
			Leave	-0.01	0.01	-0.03	0.02
			Move side	0.01	0.03	-0.05	0.08
			Submission	0.02	0.03	-0.03	0.08
**Freeloading**	472	Rank difference	No response*				
			Fight back	-0.49	0.19	-0.95	-0.18
			Leave	0.22	0.32	-0.42	0.82
			Move side	1.19	0.94	-0.40	3.33
			Submission	0.50	0.58	-0.55	1.45
		Affiliation	No response*				
			Fight back	-0.00	0.01	-0.02	0.01
			Leave	-0.01	0.01	-0.03	0.02
			Move side	-0.13	0.10	-0.39	0.01
			Submission	-0.05	0.02	-0.08	-0.02
**Monopoly**	85	Rank difference	No response*				
			Leave	-0.61	0.50	-1.59	0.39
			Move side	-0.33	1.02	-2.17	1.94
			Submission	-1.56	0.56	-2.59	-0.24
	85	Affiliation	No response*				
			Leave	-0.05	0.03	-0.11	0.00

			Move side	-0.16	0.14	-0.51	0.03
			Submission	-0.10	0.04	-0.19	-0.04
**Fight**	105	Rank difference	No response*				
			Block	4.48	6.25	-1.88	20.30
			Fight back	-2.89	2.74	-8.59	-0.36
			Leave	0.37	0.87	-1.22	2.28
			Move side	1.72	2.04	-1.03	6.64
			Submission	-0.39	0.88	-2.04	1.41
		Affiliation	No response*				
			Block	0.11	0.26	-0.23	0.79
			Fight back	0.27	0.23	0.03	0.92
			Leave	-0.02	0.02	-0.07	0.02
			Move side	-0.07	0.06	-0.20	0.03
			Submission	-0.05	0.03	-0.11	-0.00

*As reference
