# Supplementary material for: Cooperating elephants mitigate competition until the stakes get too high
Source: PLoS Biol. 2021 Sep 28;19(9):e3001391. doi: 10.1371/journal.pbio.3001391 (PMC8478180; doi:10.1371/journal.pbio.3001391)
Supplement: S7 Table — (PDF) [file pbio.3001391.s007.pdf]

S7 Table. The model results on the impacts of rank difference and affiliation on the mitigation strategies selected in the one-tray phase II. 95% CI represents 95% credible intervals.

Model (competition behavior)	n	Predictor	Response	Estimate	Std. error	Lower 95% CI	Upper 95% CI
**Approach**	202	Rank difference	No response*				
			Block	2.63	3.5	0.08	11.17
			Fight back	-0.25	0.53	-1.19	0.85
			Leave	0.28	0.25	-0.18	0.8
			Move side	1.01	0.52	0.18	2.23
			Submission	0.37	0.32	-0.14	1.12
		Affiliation	No response*				
			Block	0.12	0.18	-0.07	0.56
			Fight back	0.02	0.03	-0.03	0.08
			Leave	-0.02	0.01	-0.04	0
			Move side	-0.03	0.02	-0.08	0
			Submission	-0.03	0.01	-0.06	-0.01
**Rope pulling**	35	Rank difference	No response *				
			Fight back	0	0.67	-1.78	0.86
	35	Affiliation	No response *				
			Fight back	-0.00	0.04	-0.11	0.05
**Freeloading**	34	Rank difference	No response*				
			Block	-0.42	1.4	-3.78	1.97
			Fight back	-0.22	0.58	-1.45	0.83
			Leave	3.72	3.01	-1.25	11.14
			Submission	4.77	3.33	0.79	13.17
		Affiliation	No response*				
			Block	-0.14	0.15	-0.52	0.06
			Fight back	-0.05	0.06	-0.18	0.04
			Leave	-0.36	0.25	-1	-0.06
			Submission	-0.29	0.22	-0.84	-0.00
**Monopoly**	215	Rank difference	No response*				
			Fight back	-0.01	0.53	-0.83	1.29
			Leave	0.62	0.7	-0.36	2.41
			Move side	1.43	1.27	-0.37	4.45
			Submission	2.01	1.53	-0.04	5.86
		Affiliation	No response*				
			Fight back	0.07	0.05	-0.01	0.2
			Leave	-0.06	0.04	-0.15	-0.01

			Move side	-0.29	0.18	-0.74	-0.06
			Submission	-0.22	0.1	-0.45	-0.08
**Fight**	138	Rank difference	No response*				
			Fight back	-18.36	38.28	-125.72	-0.01
			Leave	-6.13	24.28	-70.77	16.12
			Move side	-5.98	23.31	-71.06	18.14
			Submission	2.11	2.31	-0.4	7.94
		Affiliation	No response*				
			Fight back	1.43	2.81	0.02	9.9
			Leave	1.13	2.2	-0.18	7.04
			Move side	1.15	2.08	-0.18	6.94
			Submission	-0.11	0.14	-0.46	0.07

*As reference
