# Supplementary material for: Cooperating elephants mitigate competition until the stakes get too high
Source: PLoS Biol. 2021 Sep 28;19(9):e3001391. doi: 10.1371/journal.pbio.3001391 (PMC8478180; doi:10.1371/journal.pbio.3001391)
Supplement: S8 Table — (PDF) [file pbio.3001391.s008.pdf]

S8 Table. Dominant behaviors of elephants and behavior definitions.

Dominance behaviors	Definition	Initiator is dominant	Target is dominant
**Chase away**	An elephant runs toward another elephant, and then returns to the original place (usually family unit)[1,2].	√	
**Displacement**	Rapid approach to another elephant and displacement of that elephant from any location (target must move at least one body length). No physical contact[1].	√	
**Frightened**	An elephant moves suddenly when they see another elephant, and usually moves away deliberately[2].		√
**Intervention**	Place own body between an aggressor and a target. Typically exhibited by the matriarch/dominant elephant[1].	√	
**Kicking**	Kicking at another elephant with front foot or rear foot. Kick may be projected forward with the front foot, to the side, or to the back with the back foot[1].	√	
**Play mounting**	One elephant mounts another from behind (regardless of sex). Possibly a dominant behavior, or a form of masturbation[1].	√	
**Push**	Head-to-head, head-to-body or body-to-body forceful contact that results in the	√	

	target elephant moving. Tusks are not used[1].		
**Submission**	One elephant pushes rump towards another elephant, but not for mating[1].		√
**Trunk over body**	Placing the trunk on top of another elephant's body, usually from the side[1].	√	
**Trunk over head**	Placing the trunk on top of another elephant's head, usually from the side[1].	√	
**Trunk over hip**	Placing the trunk on top of another elephant's body, usually from behind[1].	√	

[1] Definition is cited or modified from Elephant Husbandry Resource Guide-Behavior Management [75].

[2]Definition is based on routine observation.
