# Supplementary figures and images for: Cooperating elephants mitigate competition until the stakes get too high
Source: PLoS Biol. 2021 Sep 28;19(9):e3001391. doi: 10.1371/journal.pbio.3001391 (PMC8478180; doi:10.1371/journal.pbio.3001391)

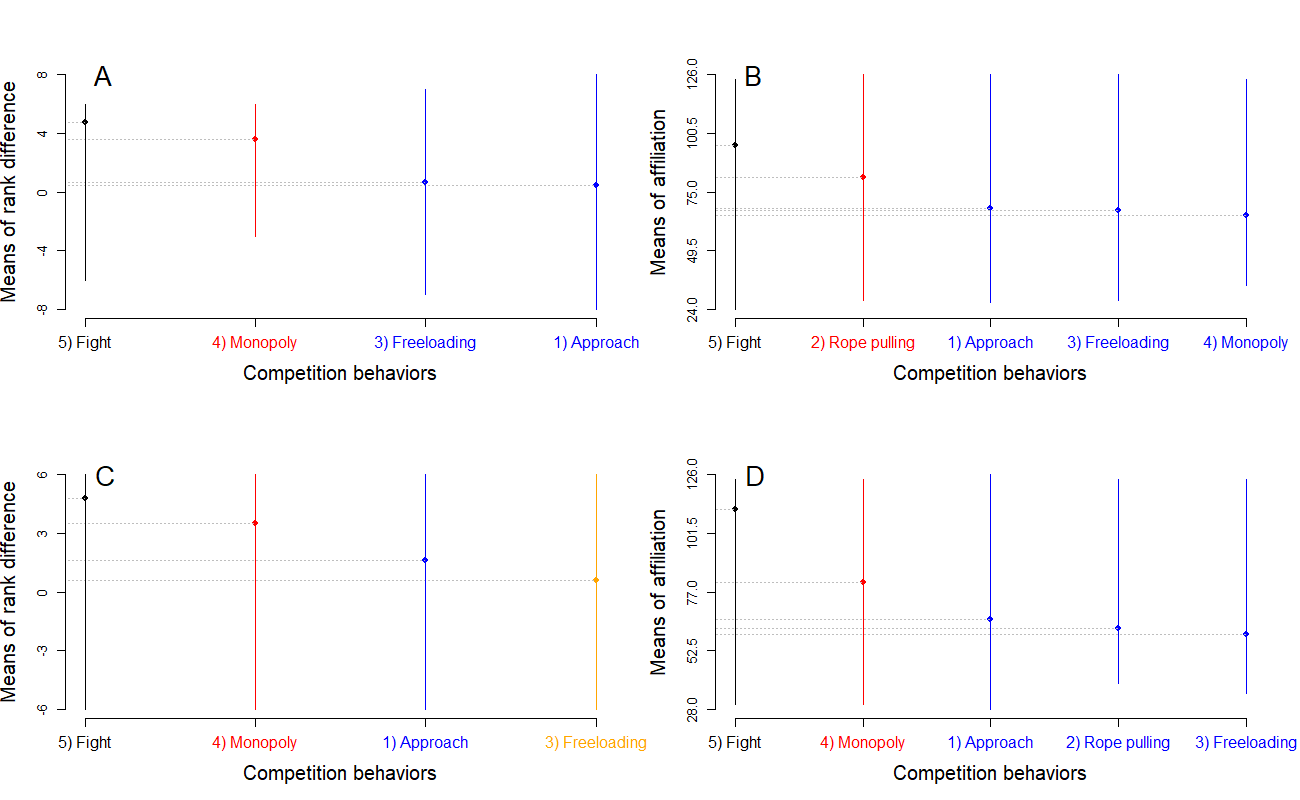

Supplement: S1 Fig — (A) Means of rank difference by competition type in 2-tray Phase I. (B) Means of affiliation by competition type in 2-tray Phase I. (C) Means of rank difference by competition type in 1-tray Phase II. (D) Means of affiliation by competition type in 1-tray Phase II. The points represent the mean values, while lines are the range of the rank difference or affiliation. Different colors represent a significant difference between behaviors, while the same color (blue) represents a nonsignificant difference. Rope pulling was not included in the means comparison of rank difference because it used a different calculation for rank difference (see Figs 3 or 5 for details). The data used to generate this figure can be found in S1 Data. (TIFF) [file pbio.3001391.s009.tiff]
